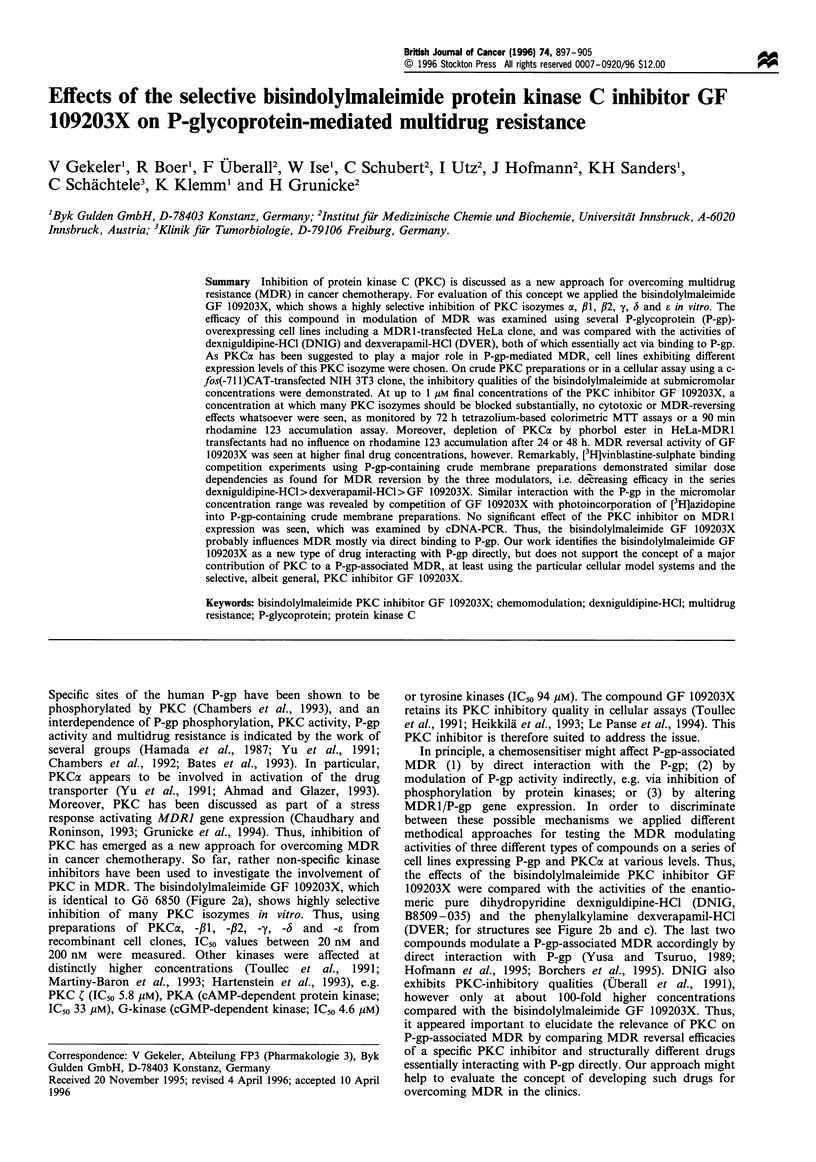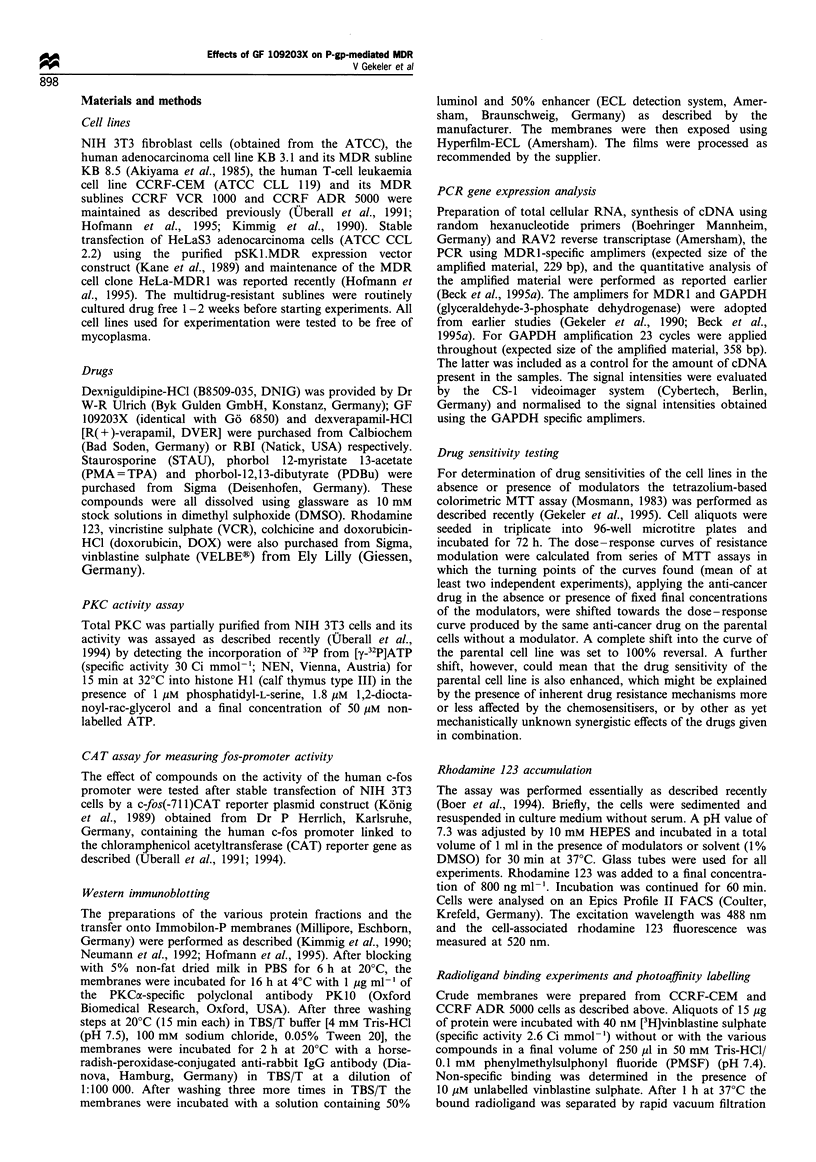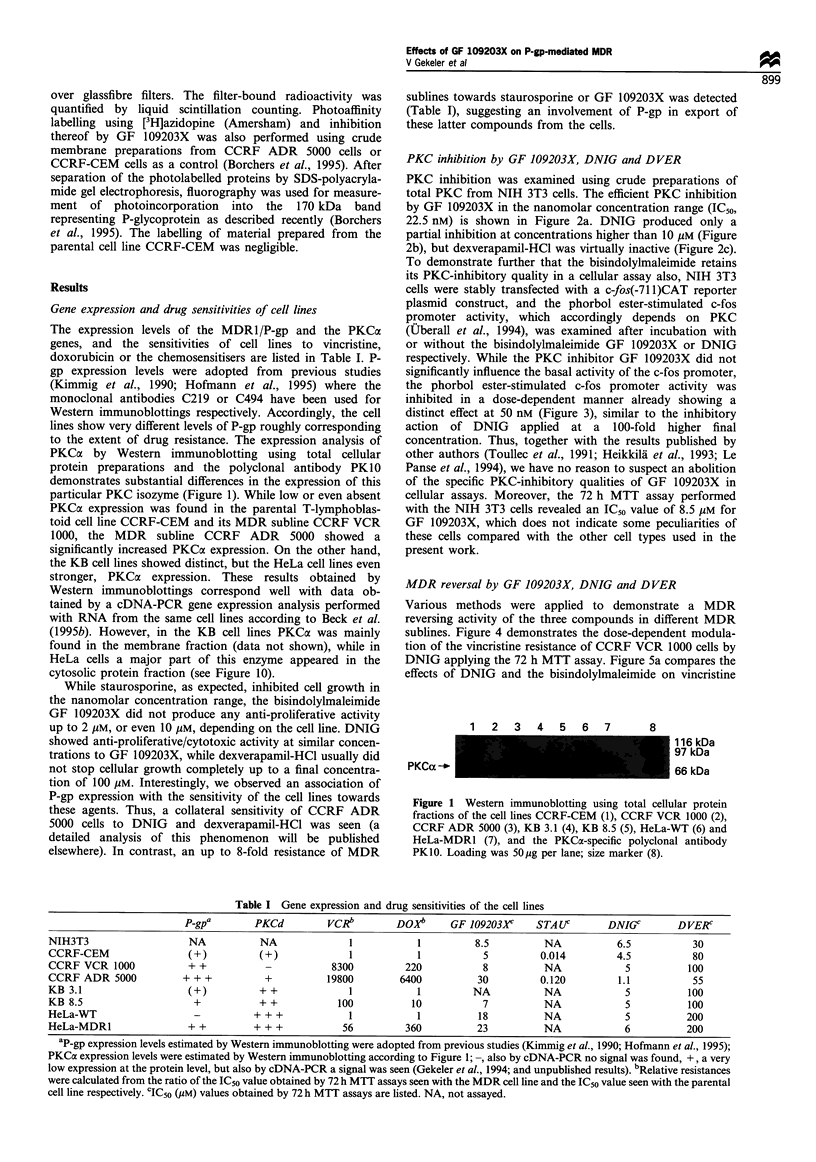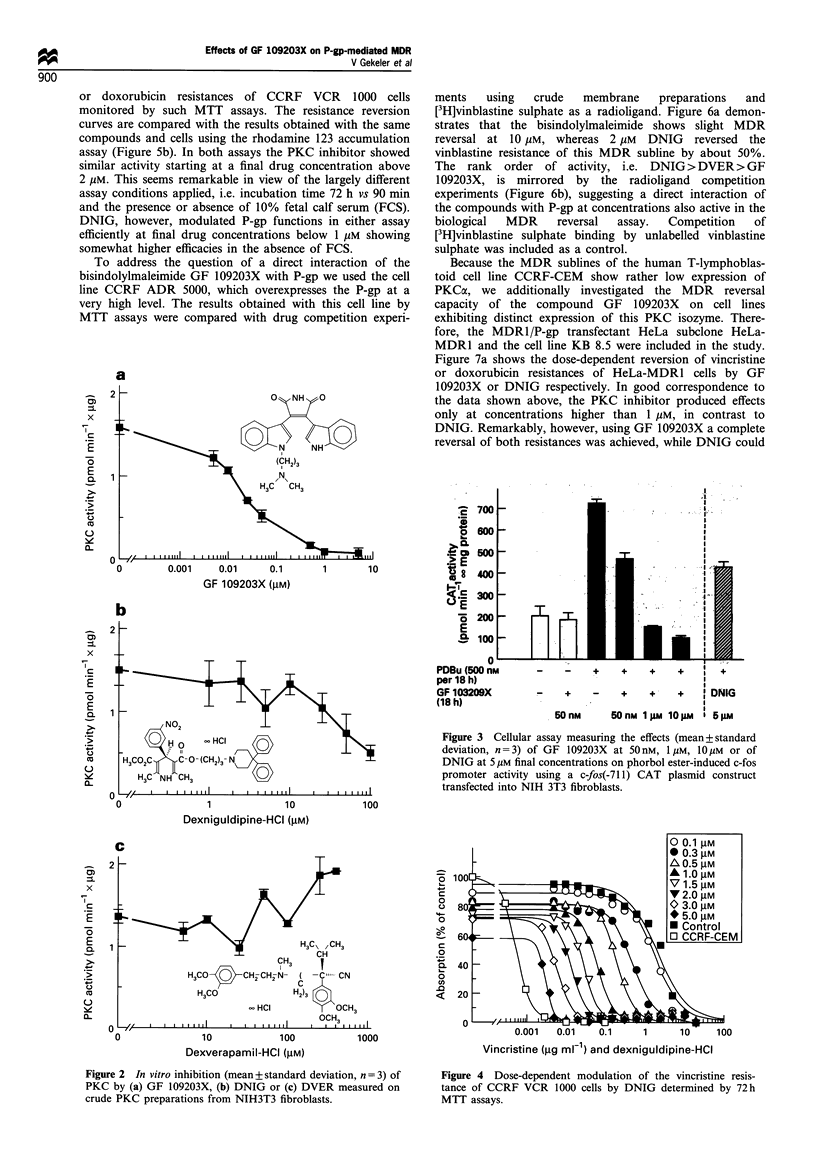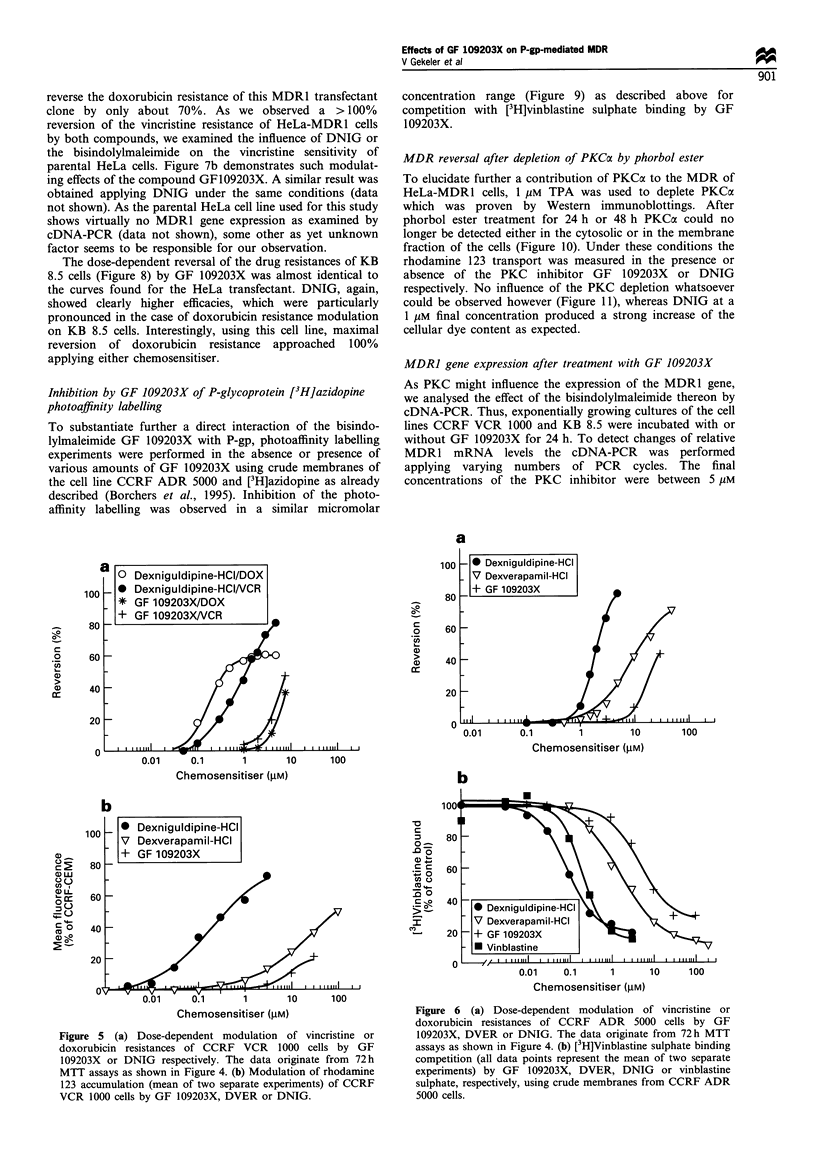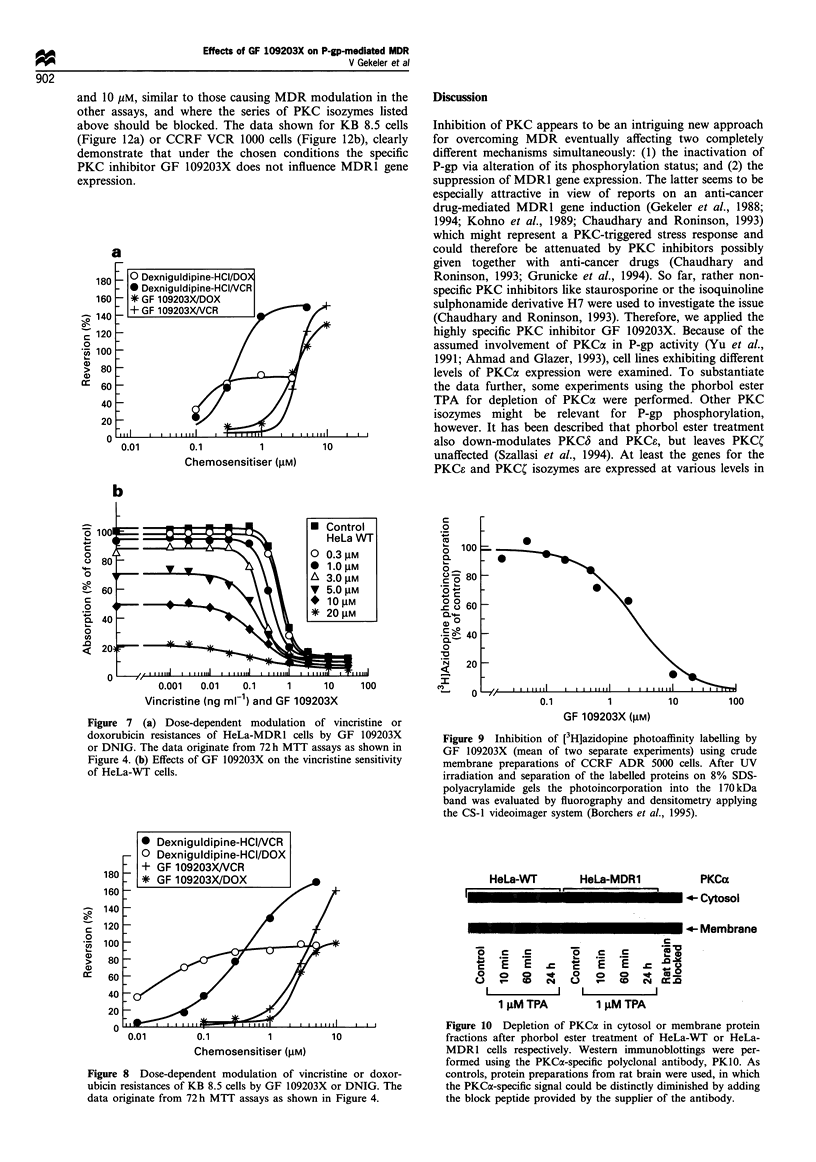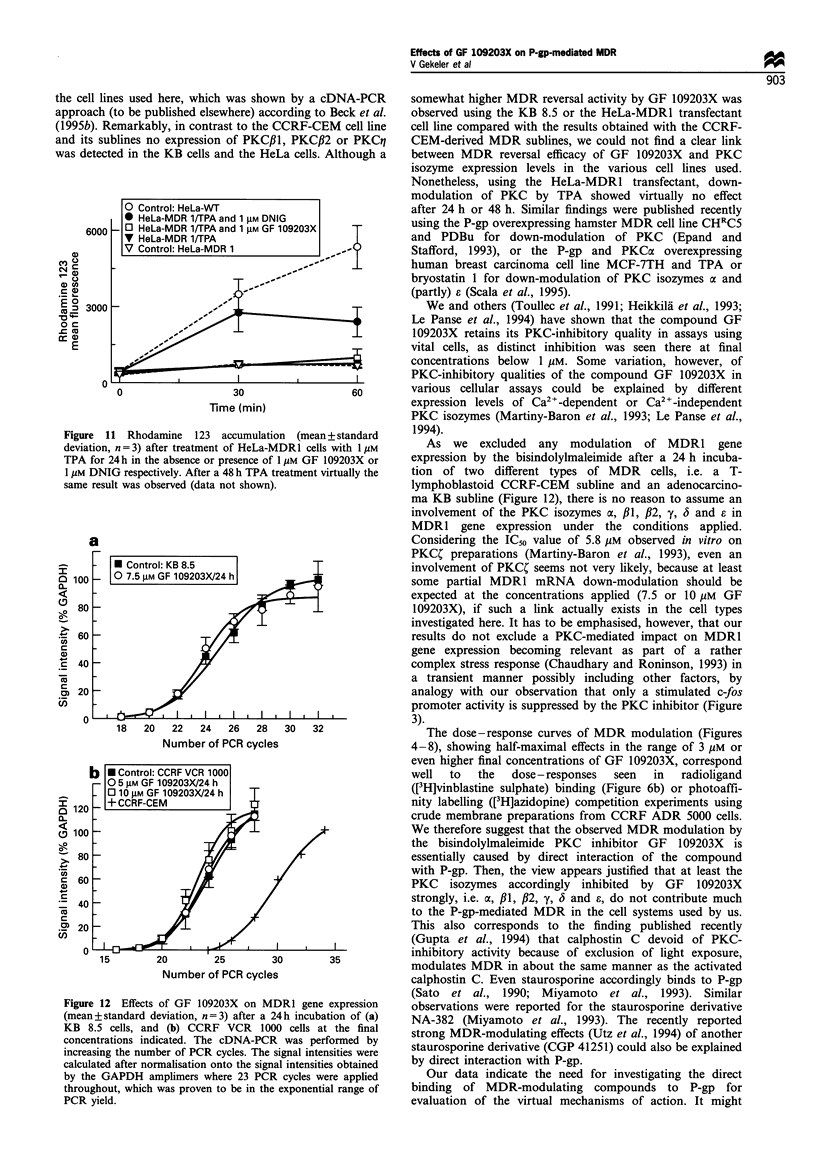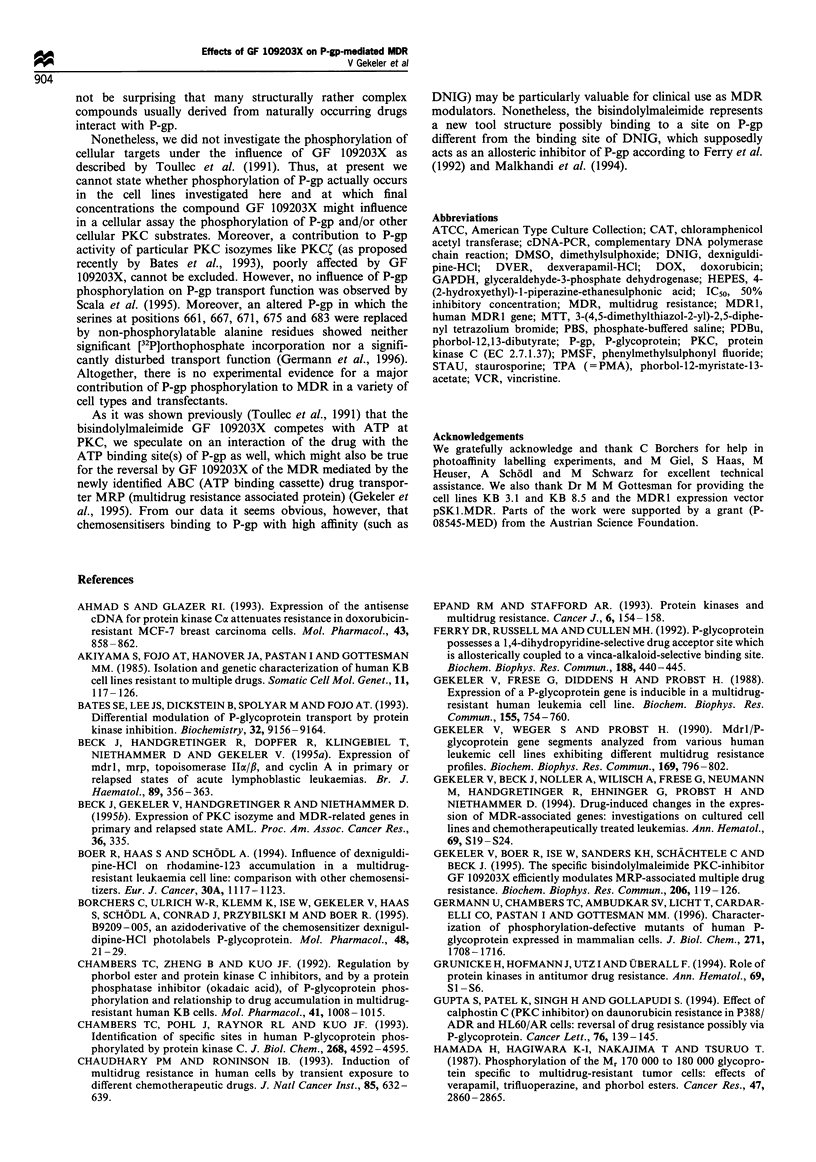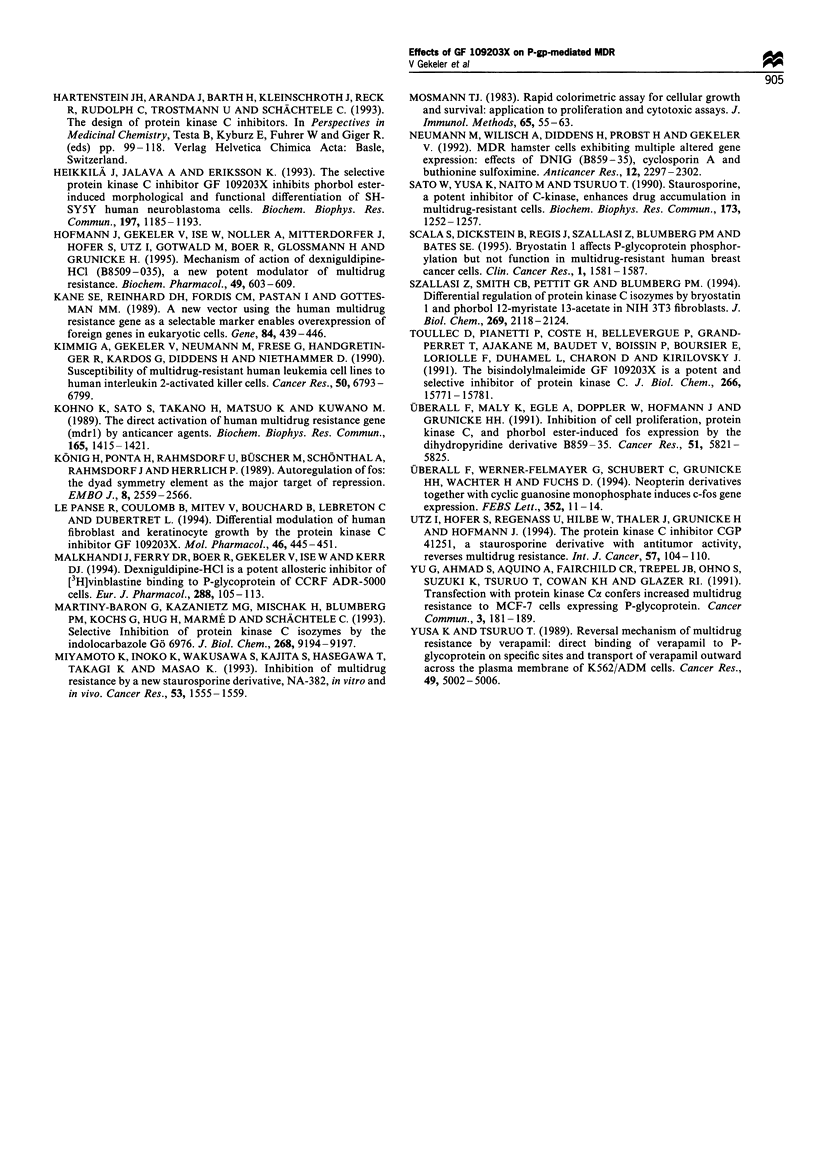# Effects of the selective bisindolylmaleimide protein kinase C inhibitor GF 109203X on P-glycoprotein-mediated multidrug resistance.

**DOI:** 10.1038/bjc.1996.454

**Published:** 1996-09

**Authors:** V. Gekeler, R. Boer, F. Uberall, W. Ise, C. Schubert, I. Utz, J. Hofmann, K. H. Sanders, C. Schächtele, K. Klemm, H. Grunicke

**Affiliations:** Byk Gulden GmbH, Konstanz, Germany.

## Abstract

**Images:**


British Journal of Cancer (1996) 74, 897-905
© 1996 Stockton Press All rights reserved 0007-0920/96 $12.00

### Effects of the selective bisindolylmaleimide protein kinase C inhibitor GF 109203X on P-glycoprotein-mediated multidrug resistance

V Gekeler[1], R Boer[1], F Überall[2], W Ise[1], C Schubert[2], I Utz[2], J Hofmann[2], KH Sanders[1], C Schächtele[3], K Klemm[1] and H Grunicke[2]

[1]*Byk Gulden GmbH, D-78403 Konstanz, Germany;* [2]*Institut für Medizinische Chemie und Biochemie, Universität Innsbruck, A-6020 Innsbruck, Austria;* [3]*Klinik für Tumorbiologie, D-79106 Freiburg, Germany.*

**Summary** Inhibition of protein kinase C (PKC) is discussed as a new approach for overcoming multidrug resistance (MDR) in cancer chemotherapy. For evaluation of this concept we applied the bisindolylmaleimide GF 109203X, which shows a highly selective inhibition of PKC isozymes $\alpha$, $\beta 1$, $\beta 2$, $\gamma$, $\delta$ and $\varepsilon$ *in vitro*. The efficacy of this compound in modulation of MDR was examined using several P-glycoprotein (P-gp)-overexpressing cell lines including a MDR1-transfected HeLa clone, and was compared with the activities of dexniguldipine-HCl (DNIG) and dexverapamil-HCl (DVER), both of which essentially act via binding to P-gp. As PKC$\alpha$ has been suggested to play a major role in P-gp-mediated MDR, cell lines exhibiting different expression levels of this PKC isozyme were chosen. On crude PKC preparations or in a cellular assay using a c-*fos*(-711)CAT-transfected NIH 3T3 clone, the inhibitory qualities of the bisindolylmaleimide at submicromolar concentrations were demonstrated. At up to 1 $\mu$M final concentrations of the PKC inhibitor GF 109203X, a concentration at which many PKC isozymes should be blocked substantially, no cytotoxic or MDR-reversing effects whatsoever were seen, as monitored by 72 h tetrazolium-based colorimetric MTT assays or a 90 min rhodamine 123 accumulation assay. Moreover, depletion of PKC$\alpha$ by phorbol ester in HeLa-MDR1 transfectants had no influence on rhodamine 123 accumulation after 24 or 48 h. MDR reversal activity of GF 109203X was seen at higher final drug concentrations, however. Remarkably, [$^3$H]vinblastine-sulphate binding competition experiments using P-gp-containing crude membrane preparations demonstrated similar dose dependencies as found for MDR reversion by the three modulators, i.e. decreasing efficacy in the series dexniguldipine-HCl > dexverapamil-HCl > GF 109203X. Similar interaction with the P-gp in the micromolar concentration range was revealed by competition of GF 109203X with photoincorporation of [$^3$H]azidopine into P-gp-containing crude membrane preparations. No significant effect of the PKC inhibitor on MDR1 expression was seen, which was examined by cDNA-PCR. Thus, the bisindolylmaleimide GF 109203X probably influences MDR mostly via direct binding to P-gp. Our work identifies the bisindolylmaleimide GF 109203X as a new type of drug interacting with P-gp directly, but does not support the concept of a major contribution of PKC to a P-gp-associated MDR, at least using the particular cellular model systems and the selective, albeit general, PKC inhibitor GF 109203X.

**Keywords:** bisindolylmaleimide PKC inhibitor GF 109203X; chemomodulation; dexniguldipine-HCl; multidrug resistance; P-glycoprotein; protein kinase C

Specific sites of the human P-gp have been shown to be phosphorylated by PKC (Chambers *et al.*, 1993), and an interdependence of P-gp phosphorylation, PKC activity, P-gp activity and multidrug resistance is indicated by the work of several groups (Hamada *et al.*, 1987; Yu *et al.*, 1991; Chambers *et al.*, 1992; Bates *et al.*, 1993). In particular, PKC$\alpha$ appears to be involved in activation of the drug transporter (Yu *et al.*, 1991; Ahmad and Glazer, 1993). Moreover, PKC has been discussed as part of a stress response activating *MDR1* gene expression (Chaudhary and Roninson, 1993; Grunicke *et al.*, 1994). Thus, inhibition of PKC has emerged as a new approach for overcoming MDR in cancer chemotherapy. So far, rather non-specific kinase inhibitors have been used to investigate the involvement of PKC in MDR. The bisindolylmaleimide GF 109203X, which is identical to Gö 6850 (Figure 2a), shows highly selective inhibition of many PKC isozymes *in vitro*. Thus, using preparations of PKC$\alpha$, -$\beta 1$, -$\beta 2$, -$\gamma$, -$\delta$ and -$\varepsilon$ from recombinant cell clones, $IC_{50}$ values between 20 nM and 200 nM were measured. Other kinases were affected at distinctly higher concentrations (Toullec *et al.*, 1991; Martiny-Baron *et al.*, 1993; Hartenstein *et al.*, 1993), e.g. PKC $\zeta$ ($IC_{50}$ 5.8 $\mu$M), PKA (cAMP-dependent protein kinase; $IC_{50}$ 33 $\mu$M), G-kinase (cGMP-dependent kinase; $IC_{50}$ 4.6 $\mu$M) or tyrosine kinases ($IC_{50}$ 94 $\mu$M). The compound GF 109203X retains its PKC inhibitory quality in cellular assays (Toullec *et al.*, 1991; Heikkilä *et al.*, 1993; Le Panse *et al.*, 1994). This PKC inhibitor is therefore suited to address the issue.

In principle, a chemosensitiser might affect P-gp-associated MDR (1) by direct interaction with the P-gp; (2) by modulation of P-gp activity indirectly, e.g. via inhibition of phosphorylation by protein kinases; or (3) by altering MDR1/P-gp gene expression. In order to discriminate between these possible mechanisms we applied different methodical approaches for testing the MDR modulating activities of three different types of compounds on a series of cell lines expressing P-gp and PKC$\alpha$ at various levels. Thus, the effects of the bisindolylmaleimide PKC inhibitor GF 109203X were compared with the activities of the enantiomeric pure dihydropyridine dexniguldipine-HCl (DNIG, B8509-035) and the phenylalkylamine dexverapamil-HCl (DVER; for structures see Figure 2b and c). The last two compounds modulate a P-gp-associated MDR accordingly by direct interaction with P-gp (Yusa and Tsuruo, 1989; Hofmann *et al.*, 1995; Borchers *et al.*, 1995). DNIG also exhibits PKC-inhibitory qualities (Überall *et al.*, 1991), however only at about 100-fold higher concentrations compared with the bisindolylmaleimide GF 109203X. Thus, it appeared important to elucidate the relevance of PKC on P-gp-associated MDR by comparing MDR reversal efficacies of a specific PKC inhibitor and structurally different drugs essentially interacting with P-gp directly. Our approach might help to evaluate the concept of developing such drugs for overcoming MDR in the clinics.

Correspondence: V Gekeler, Abteilung FP3 (Pharmakologie 3), Byk Gulden GmbH, D-78403 Konstanz, Germany
Received 20 November 1995; revised 4 April 1996; accepted 10 April 1996

### Materials and methods

*Cell lines*

NIH 3T3 fibroblast cells (obtained from the ATCC), the human adenocarcinoma cell line KB 3.1 and its MDR subline KB 8.5 (Akiyama *et al.*, 1985), the human T-cell leukaemia cell line CCRF-CEM (ATCC CLL 119) and its MDR sublines CCRF VCR 1000 and CCRF ADR 5000 were maintained as described previously (Überall *et al.*, 1991; Hofmann *et al.*, 1995; Kimmig *et al.*, 1990). Stable transfection of HeLaS3 adenocarcinoma cells (ATCC CCL 2.2) using the purified pSK1.MDR expression vector construct (Kane *et al.*, 1989) and maintenance of the MDR cell clone HeLa-MDR1 was reported recently (Hofmann *et al.*, 1995). The multidrug-resistant sublines were routinely cultured drug free 1–2 weeks before starting experiments. All cell lines used for experimentation were tested to be free of mycoplasma.

*Drugs*

Dexniguldipine-HCl (B8509-035, DNIG) was provided by Dr W-R Ulrich (Byk Gulden GmbH, Konstanz, Germany); GF 109203X (identical with Gö 6850) and dexverapamil-HCl [R(+)-verapamil, DVER] were purchased from Calbiochem (Bad Soden, Germany) or RBI (Natick, USA) respectively. Staurosporine (STAU), phorbol 12-myristate 13-acetate (PMA=TPA) and phorbol-12,13-dibutyrate (PDBu) were purchased from Sigma (Deisenhofen, Germany). These compounds were all dissolved using glassware as 10 mM stock solutions in dimethyl sulphoxide (DMSO). Rhodamine 123, vincristine sulphate (VCR), colchicine and doxorubicin-HCl (doxorubicin, DOX) were also purchased from Sigma, vinblastine sulphate (VELBE®) from Ely Lilly (Giessen, Germany).

*PKC activity assay*

Total PKC was partially purified from NIH 3T3 cells and its activity was assayed as described recently (Überall *et al.*, 1994) by detecting the incorporation of $^{32}P$ from [γ-$^{32}P$]ATP (specific activity 30 Ci $mmol^{-1}$; NEN, Vienna, Austria) for 15 min at 32°C into histone H1 (calf thymus type III) in the presence of 1 μM phosphatidyl-L-serine, 1.8 μM 1,2-dioctanoyl-rac-glycerol and a final concentration of 50 μM non-labelled ATP.

*CAT assay for measuring fos-promoter activity*

The effect of compounds on the activity of the human c-fos promoter were tested after stable transfection of NIH 3T3 cells by a c-*fos*(-711)CAT reporter plasmid construct (König *et al.*, 1989) obtained from Dr P Herrlich, Karlsruhe, Germany, containing the human c-fos promoter linked to the chloramphenicol acetyltransferase (CAT) reporter gene as described (Überall *et al.*, 1991; 1994).

*Western immunoblotting*

The preparations of the various protein fractions and the transfer onto Immobilon-P membranes (Millipore, Eschborn, Germany) were performed as described (Kimmig *et al.*, 1990; Neumann *et al.*, 1992; Hofmann *et al.*, 1995). After blocking with 5% non-fat dried milk in PBS for 6 h at 20°C, the membranes were incubated for 16 h at 4°C with 1 μg $ml^{-1}$ of the PKCα-specific polyclonal antibody PK10 (Oxford Biomedical Research, Oxford, USA). After three washing steps at 20°C (15 min each) in TBS/T buffer [4 mM Tris-HCl (pH 7.5), 100 mM sodium chloride, 0.05% Tween 20], the membranes were incubated for 2 h at 20°C with a horse-radish-peroxidase-conjugated anti-rabbit IgG antibody (Dianova, Hamburg, Germany) in TBS/T at a dilution of 1:100 000. After washing three more times in TBS/T the membranes were incubated with a solution containing 50% luminol and 50% enhancer (ECL detection system, Amersham, Braunschweig, Germany) as described by the manufacturer. The membranes were then exposed using Hyperfilm-ECL (Amersham). The films were processed as recommended by the supplier.

*PCR gene expression analysis*

Preparation of total cellular RNA, synthesis of cDNA using random hexanucleotide primers (Boehringer Mannheim, Germany) and RAV2 reverse transcriptase (Amersham), the PCR using MDR1-specific amplimers (expected size of the amplified material, 229 bp), and the quantitative analysis of the amplified material were performed as reported earlier (Beck *et al.*, 1995*a*). The amplimers for MDR1 and GAPDH (glyceraldehyde-3-phosphate dehydrogenase) were adopted from earlier studies (Gekeler *et al.*, 1990; Beck *et al.*, 1995*a*). For GAPDH amplification 23 cycles were applied throughout (expected size of the amplified material, 358 bp). The latter was included as a control for the amount of cDNA present in the samples. The signal intensities were evaluated by the CS-1 videoimager system (Cybertech, Berlin, Germany) and normalised to the signal intensities obtained using the GAPDH specific amplimers.

*Drug sensitivity testing*

For determination of drug sensitivities of the cell lines in the absence or presence of modulators the tetrazolium-based colorimetric MTT assay (Mosmann, 1983) was performed as described recently (Gekeler *et al.*, 1995). Cell aliquots were seeded in triplicate into 96-well microtitre plates and incubated for 72 h. The dose–response curves of resistance modulation were calculated from series of MTT assays in which the turning points of the curves found (mean of at least two independent experiments), applying the anti-cancer drug in the absence or presence of fixed final concentrations of the modulators, were shifted towards the dose–response curve produced by the same anti-cancer drug on the parental cells without a modulator. A complete shift into the curve of the parental cell line was set to 100% reversal. A further shift, however, could mean that the drug sensitivity of the parental cell line is also enhanced, which might be explained by the presence of inherent drug resistance mechanisms more or less affected by the chemosensitisers, or by other as yet mechanistically unknown synergistic effects of the drugs given in combination.

*Rhodamine 123 accumulation*

The assay was performed essentially as described recently (Boer *et al.*, 1994). Briefly, the cells were sedimented and resuspended in culture medium without serum. A pH value of 7.3 was adjusted by 10 mM HEPES and incubated in a total volume of 1 ml in the presence of modulators or solvent (1% DMSO) for 30 min at 37°C. Glass tubes were used for all experiments. Rhodamine 123 was added to a final concentration of 800 ng $ml^{-1}$. Incubation was continued for 60 min. Cells were analysed on an Epics Profile II FACS (Coulter, Krefeld, Germany). The excitation wavelength was 488 nm and the cell-associated rhodamine 123 fluorescence was measured at 520 nm.

*Radioligand binding experiments and photoaffinity labelling*

Crude membranes were prepared from CCRF-CEM and CCRF ADR 5000 cells as described above. Aliquots of 15 μg of protein were incubated with 40 nM [$^3H$]vinblastine sulphate (specific activity 2.6 Ci $mmol^{-1}$) without or with the various compounds in a final volume of 250 μl in 50 mM Tris-HCl/0.1 mM phenylmethylsulphonyl fluoride (PMSF) (pH 7.4). Non-specific binding was determined in the presence of 10 μM unlabelled vinblastine sulphate. After 1 h at 37°C the bound radioligand was separated by rapid vacuum filtration over glassfibre filters. The filter-bound radioactivity was quantified by liquid scintillation counting. Photoaffinity labelling using [$^3$H]azidopine (Amersham) and inhibition thereof by GF 109203X was also performed using crude membrane preparations from CCRF ADR 5000 cells or CCRF-CEM cells as a control (Borchers *et al.*, 1995). After separation of the photolabelled proteins by SDS-polyacrylamide gel electrophoresis, fluorography was used for measurement of photoincorporation into the 170 kDa band representing P-glycoprotein as described recently (Borchers *et al.*, 1995). The labelling of material prepared from the parental cell line CCRF-CEM was negligible.

### Results

### *Gene expression and drug sensitivities of cell lines*

The expression levels of the MDR1/P-gp and the PKCα genes, and the sensitivities of cell lines to vincristine, doxorubicin or the chemosensitisers are listed in Table I. P-gp expression levels were adopted from previous studies (Kimmig *et al.*, 1990; Hofmann *et al.*, 1995) where the monoclonal antibodies C219 or C494 have been used for Western immunoblottings respectively. Accordingly, the cell lines show very different levels of P-gp roughly corresponding to the extent of drug resistance. The expression analysis of PKCα by Western immunoblotting using total cellular protein preparations and the polyclonal antibody PK10 demonstrates substantial differences in the expression of this particular PKC isozyme (Figure 1). While low or even absent PKCα expression was found in the parental T-lymphoblastoid cell line CCRF-CEM and its MDR subline CCRF VCR 1000, the MDR subline CCRF ADR 5000 showed a significantly increased PKCα expression. On the other hand, the KB cell lines showed distinct, but the HeLa cell lines even stronger, PKCα expression. These results obtained by Western immunoblottings correspond well with data obtained by a cDNA-PCR gene expression analysis performed with RNA from the same cell lines according to Beck *et al.* (1995*b*). However, in the KB cell lines PKCα was mainly found in the membrane fraction (data not shown), while in HeLa cells a major part of this enzyme appeared in the cytosolic protein fraction (see Figure 10).

While staurosporine, as expected, inhibited cell growth in the nanomolar concentration range, the bisindolylmaleimide GF 109203X did not produce any anti-proliferative activity up to 2 μM, or even 10 μM, depending on the cell line. DNIG showed anti-proliferative/cytotoxic activity at similar concentrations to GF 109203X, while dexverapamil-HCl usually did not stop cellular growth completely up to a final concentration of 100 μM. Interestingly, we observed an association of P-gp expression with the sensitivity of the cell lines towards these agents. Thus, a collateral sensitivity of CCRF ADR 5000 cells to DNIG and dexverapamil-HCl was seen (a detailed analysis of this phenomenon will be published elsewhere). In contrast, an up to 8-fold resistance of MDR sublines towards staurosporine or GF 109203X was detected (Table I), suggesting an involvement of P-gp in export of these latter compounds from the cells.

### *PKC inhibition by GF 109203X, DNIG and DVER*

PKC inhibition was examined using crude preparations of total PKC from NIH 3T3 cells. The efficient PKC inhibition by GF 109203X in the nanomolar concentration range ($IC_{50}$, 22.5 nM) is shown in Figure 2a. DNIG produced only a partial inhibition at concentrations higher than 10 μM (Figure 2b), but dexverapamil-HCl was virtually inactive (Figure 2c). To demonstrate further that the bisindolylmaleimide retains its PKC-inhibitory quality in a cellular assay also, NIH 3T3 cells were stably transfected with a c-*fos*(-711)CAT reporter plasmid construct, and the phorbol ester-stimulated c-fos promoter activity, which accordingly depends on PKC (Überall *et al.*, 1994), was examined after incubation with or without the bisindolylmaleimide GF 109203X or DNIG respectively. While the PKC inhibitor GF 109203X did not significantly influence the basal activity of the c-fos promoter, the phorbol ester-stimulated c-fos promoter activity was inhibited in a dose-dependent manner already showing a distinct effect at 50 nM (Figure 3), similar to the inhibitory action of DNIG applied at a 100-fold higher final concentration. Thus, together with the results published by other authors (Toullec *et al.*, 1991; Heikkilä *et al.*, 1993; Le Panse *et al.*, 1994), we have no reason to suspect an abolition of the specific PKC-inhibitory qualities of GF 109203X in cellular assays. Moreover, the 72 h MTT assay performed with the NIH 3T3 cells revealed an $IC_{50}$ value of 8.5 μM for GF 109203X, which does not indicate some peculiarities of these cells compared with the other cell types used in the present work.

### *MDR reversal by GF 109203X, DNIG and DVER*

Various methods were applied to demonstrate a MDR reversing activity of the three compounds in different MDR sublines. Figure 4 demonstrates the dose-dependent modulation of the vincristine resistance of CCRF VCR 1000 cells by DNIG applying the 72 h MTT assay. Figure 5a compares the effects of DNIG and the bisindolylmaleimide on vincristine

**Figure 1** Western immunoblotting using total cellular protein fractions of the cell lines CCRF-CEM (1), CCRF VCR 1000 (2), CCRF ADR 5000 (3), KB 3.1 (4), KB 8.5 (5), HeLa-WT (6) and HeLa-MDR1 (7), and the PKCα-specific polyclonal antibody PK10. Loading was 50 μg per lane; size marker (8).

**Table I** Gene expression and drug sensitivities of the cell lines

| | *P-gp*[a] | *PKCd* | *VCR*[b] | *DOX*[b] | *GF 109203X*[c] | *STAU*[c] | *DNIG*[c] | *DVER*[c] |
|---|---|---|---|---|---|---|---|---|
| NIH3T3 | NA | NA | 1 | 1 | 8.5 | NA | 6.5 | 30 |
| CCRF-CEM | (+) | (+) | 1 | 1 | 5 | 0.014 | 4.5 | 80 |
| CCRF VCR 1000 | + + | – | 8300 | 220 | 8 | NA | 5 | 100 |
| CCRF ADR 5000 | + + + | + | 19800 | 6400 | 30 | 0.120 | 1.1 | 55 |
| KB 3.1 | (+) | + + | 1 | 1 | NA | NA | 5 | 100 |
| KB 8.5 | + | + + | 100 | 10 | 7 | NA | 5 | 100 |
| HeLa-WT | – | + + + | 1 | 1 | 18 | NA | 5 | 200 |
| HeLa-MDR1 | + + | + + + | 56 | 360 | 23 | NA | 6 | 200 |

[a]P-gp expression levels estimated by Western immunoblotting were adopted from previous studies (Kimmig *et al.*, 1990; Hofmann *et al.*, 1995); PKCα expression levels were estimated by Western immunoblotting according to Figure 1; –, also by cDNA-PCR no signal was found, +, a very low expression at the protein level, but also by cDNA-PCR a signal was seen (Gekeler *et al.*, 1994; and unpublished results). [b]Relative resistances were calculated from the ratio of the $IC_{50}$ value obtained by 72 h MTT assays seen with the MDR cell line and the $IC_{50}$ value seen with the parental cell line respectively. [c]$IC_{50}$ (μM) values obtained by 72 h MTT assays are listed. NA, not assayed.

or doxorubicin resistances of CCRF VCR 1000 cells monitored by such MTT assays. The resistance reversion curves are compared with the results obtained with the same compounds and cells using the rhodamine 123 accumulation assay (Figure 5b). In both assays the PKC inhibitor showed similar activity starting at a final drug concentration above 2 $\mu$M. This seems remarkable in view of the largely different assay conditions applied, i.e. incubation time 72 h *vs* 90 min and the presence or absence of 10% fetal calf serum (FCS). DNIG, however, modulated P-gp functions in either assay efficiently at final drug concentrations below 1 $\mu$M showing somewhat higher efficacies in the absence of FCS.

To address the question of a direct interaction of the bisindolylmaleimide GF 109203X with P-gp we used the cell line CCRF ADR 5000, which overexpresses the P-gp at a very high level. The results obtained with this cell line by MTT assays were compared with drug competition experiments using crude membrane preparations and [$^3$H]vinblastine sulphate as a radioligand. Figure 6a demonstrates that the bisindolylmaleimide shows slight MDR reversal at 10 $\mu$M, whereas 2 $\mu$M DNIG reversed the vinblastine resistance of this MDR subline by about 50%. The rank order of activity, i.e. DNIG > DVER > GF 109203X, is mirrored by the radioligand competition experiments (Figure 6b), suggesting a direct interaction of the compounds with P-gp at concentrations also active in the biological MDR reversal assay. Competition of [$^3$H]vinblastine sulphate binding by unlabelled vinblastine sulphate was included as a control.

Because the MDR sublines of the human T-lymphoblastoid cell line CCRF-CEM show rather low expression of PKC$\alpha$, we additionally investigated the MDR reversal capacity of the compound GF 109203X on cell lines exhibiting distinct expression of this PKC isozyme. Therefore, the MDR1/P-gp transfectant HeLa subclone HeLa-MDR1 and the cell line KB 8.5 were included in the study. Figure 7a shows the dose-dependent reversion of vincristine or doxorubicin resistances of HeLa-MDR1 cells by GF 109203X or DNIG respectively. In good correspondence to the data shown above, the PKC inhibitor produced effects only at concentrations higher than 1 $\mu$M, in contrast to DNIG. Remarkably, however, using GF 109203X a complete reversal of both resistances was achieved, while DNIG could

**Figure 2** *In vitro* inhibition (mean ± standard deviation, $n = 3$) of PKC by (**a**) GF 109203X, (**b**) DNIG or (**c**) DVER measured on crude PKC preparations from NIH3T3 fibroblasts.

**Figure 3** Cellular assay measuring the effects (mean ± standard deviation, $n = 3$) of GF 109203X at 50 nM, 1 $\mu$M, 10 $\mu$M or of DNIG at 5 $\mu$M final concentrations on phorbol ester-induced c-fos promoter activity using a c-*fos*(-711) CAT plasmid construct transfected into NIH 3T3 fibroblasts.

**Figure 4** Dose-dependent modulation of the vincristine resistance of CCRF VCR 1000 cells by DNIG determined by 72 h MTT assays.

reverse the doxorubicin resistance of this MDR1 transfectant clone by only about 70%. As we observed a >100% reversion of the vincristine resistance of HeLa-MDR1 cells by both compounds, we examined the influence of DNIG or the bisindolylmaleimide on the vincristine sensitivity of parental HeLa cells. Figure 7b demonstrates such modulating effects of the compound GF109203X. A similar result was obtained applying DNIG under the same conditions (data not shown). As the parental HeLa cell line used for this study shows virtually no MDR1 gene expression as examined by cDNA-PCR (data not shown), some other as yet unknown factor seems to be responsible for our observation.

The dose-dependent reversal of the drug resistances of KB 8.5 cells (Figure 8) by GF 109203X was almost identical to the curves found for the HeLa transfectant. DNIG, again, showed clearly higher efficacies, which were particularly pronounced in the case of doxorubicin resistance modulation on KB 8.5 cells. Interestingly, using this cell line, maximal reversion of doxorubicin resistance approached 100% applying either chemosensitiser.

### *Inhibition by GF 109203X of P-glycoprotein [³H]azidopine photoaffinity labelling*

To substantiate further a direct interaction of the bisindolylmaleimide GF 109203X with P-gp, photoaffinity labelling experiments were performed in the absence or presence of various amounts of GF 109203X using crude membranes of the cell line CCRF ADR 5000 and [$^3$H]azidopine as already described (Borchers *et al.*, 1995). Inhibition of the photoaffinity labelling was observed in a similar micromolar concentration range (Figure 9) as described above for competition with [$^3$H]vinblastine sulphate binding by GF 109203X.

### *MDR reversal after depletion of PKCα by phorbol ester*

To elucidate further a contribution of PKCα to the MDR of HeLa-MDR1 cells, 1 μM TPA was used to deplete PKCα which was proven by Western immunoblottings. After phorbol ester treatment for 24 h or 48 h PKCα could no longer be detected either in the cytosolic or in the membrane fraction of the cells (Figure 10). Under these conditions the rhodamine 123 transport was measured in the presence or absence of the PKC inhibitor GF 109203X or DNIG respectively. No influence of the PKC depletion whatsoever could be observed however (Figure 11), whereas DNIG at a 1 μM final concentration produced a strong increase of the cellular dye content as expected.

### *MDR1 gene expression after treatment with GF 109203X*

As PKC might influence the expression of the MDR1 gene, we analysed the effect of the bisindolylmaleimide thereon by cDNA-PCR. Thus, exponentially growing cultures of the cell lines CCRF VCR 1000 and KB 8.5 were incubated with or without GF 109203X for 24 h. To detect changes of relative MDR1 mRNA levels the cDNA-PCR was performed applying varying numbers of PCR cycles. The final concentrations of the PKC inhibitor were between 5 μM

**Figure 5** (**a**) Dose-dependent modulation of vincristine or doxorubicin resistances of CCRF VCR 1000 cells by GF 109203X or DNIG respectively. The data originate from 72 h MTT assays as shown in Figure 4. (**b**) Modulation of rhodamine 123 accumulation (mean of two separate experiments) of CCRF VCR 1000 cells by GF 109203X, DVER or DNIG.

**Figure 6** (**a**) Dose-dependent modulation of vincristine or doxorubicin resistances of CCRF ADR 5000 cells by GF 109203X, DVER or DNIG. The data originate from 72 h MTT assays as shown in Figure 4. (**b**) [$^3$H]Vinblastine sulphate binding competition (all data points represent the mean of two separate experiments) by GF 109203X, DVER, DNIG or vinblastine sulphate, respectively, using crude membranes from CCRF ADR 5000 cells.

and 10 μM, similar to those causing MDR modulation in the other assays, and where the series of PKC isozymes listed above should be blocked. The data shown for KB 8.5 cells (Figure 12a) or CCRF VCR 1000 cells (Figure 12b), clearly demonstrate that under the chosen conditions the specific PKC inhibitor GF 109203X does not influence MDR1 gene expression.

**Figure 7** (**a**) Dose-dependent modulation of vincristine or doxorubicin resistances of HeLa-MDR1 cells by GF 109203X or DNIG. The data originate from 72 h MTT assays as shown in Figure 4. (**b**) Effects of GF 109203X on the vincristine sensitivity of HeLa-WT cells.

**Figure 8** Dose-dependent modulation of vincristine or doxorubicin resistances of KB 8.5 cells by GF 109203X or DNIG. The data originate from 72 h MTT assays as shown in Figure 4.

### Discussion

Inhibition of PKC appears to be an intriguing new approach for overcoming MDR eventually affecting two completely different mechanisms simultaneously: (1) the inactivation of P-gp via alteration of its phosphorylation status; and (2) the suppression of MDR1 gene expression. The latter seems to be especially attractive in view of reports on an anti-cancer drug-mediated MDR1 gene induction (Gekeler *et al.*, 1988; 1994; Kohno *et al.*, 1989; Chaudhary and Roninson, 1993) which might represent a PKC-triggered stress response and could therefore be attenuated by PKC inhibitors possibly given together with anti-cancer drugs (Chaudhary and Roninson, 1993; Grunicke *et al.*, 1994). So far, rather non-specific PKC inhibitors like staurosporine or the isoquinoline sulphonamide derivative H7 were used to investigate the issue (Chaudhary and Roninson, 1993). Therefore, we applied the highly specific PKC inhibitor GF 109203X. Because of the assumed involvement of PKCα in P-gp activity (Yu *et al.*, 1991; Ahmad and Glazer, 1993), cell lines exhibiting different levels of PKCα expression were examined. To substantiate the data further, some experiments using the phorbol ester TPA for depletion of PKCα were performed. Other PKC isozymes might be relevant for P-gp phosphorylation, however. It has been described that phorbol ester treatment also down-modulates PKCδ and PKCε, but leaves PKCζ unaffected (Szallasi *et al.*, 1994). At least the genes for the PKCε and PKCζ isozymes are expressed at various levels in

**Figure 9** Inhibition of [$^3$H]azidopine photoaffinity labelling by GF 109203X (mean of two separate experiments) using crude membrane preparations of CCRF ADR 5000 cells. After UV irradiation and separation of the labelled proteins on 8% SDS-polyacrylamide gels the photoincorporation into the 170 kDa band was evaluated by fluorography and densitometry applying the CS-1 videoimager system (Borchers *et al.*, 1995).

**Figure 10** Depletion of PKCα in cytosol or membrane protein fractions after phorbol ester treatment of HeLa-WT or HeLa-MDR1 cells respectively. Western immunoblottings were performed using the PKCα-specific polyclonal antibody, PK10. As controls, protein preparations from rat brain were used, in which the PKCα-specific signal could be distinctly diminished by adding the block peptide provided by the supplier of the antibody.

the cell lines used here, which was shown by a cDNA-PCR approach (to be published elsewhere) according to Beck *et al.* (1995*b*). Remarkably, in contrast to the CCRF-CEM cell line and its sublines no expression of PKC$\beta$1, PKC$\beta$2 or PKC$\eta$ was detected in the KB cells and the HeLa cells. Although a somewhat higher MDR reversal activity by GF 109203X was observed using the KB 8.5 or the HeLa-MDR1 transfectant cell line compared with the results obtained with the CCRF-CEM-derived MDR sublines, we could not find a clear link between MDR reversal efficacy of GF 109203X and PKC isozyme expression levels in the various cell lines used. Nonetheless, using the HeLa-MDR1 transfectant, down-modulation of PKC by TPA showed virtually no effect after 24 h or 48 h. Similar findings were published recently using the P-gp overexpressing hamster MDR cell line $CH^RC5$ and PDBu for down-modulation of PKC (Epand and Stafford, 1993), or the P-gp and PKC$\alpha$ overexpressing human breast carcinoma cell line MCF-7TH and TPA or bryostatin 1 for down-modulation of PKC isozymes $\alpha$ and (partly) $\varepsilon$ (Scala *et al.*, 1995).

**Figure 11** Rhodamine 123 accumulation (mean $\pm$ standard deviation, $n=3$) after treatment of HeLa-MDR1 cells with 1 $\mu$M TPA for 24 h in the absence or presence of 1 $\mu$M GF 109203X or 1 $\mu$M DNIG respectively. After a 48 h TPA treatment virtually the same result was observed (data not shown).

We and others (Toullec *et al.*, 1991; Heikkilä *et al.*, 1993; Le Panse *et al.*, 1994) have shown that the compound GF 109203X retains its PKC-inhibitory quality in assays using vital cells, as distinct inhibition was seen there at final concentrations below 1 $\mu$M. Some variation, however, of PKC-inhibitory qualities of the compound GF 109203X in various cellular assays could be explained by different expression levels of $Ca^{2+}$-dependent or $Ca^{2+}$-independent PKC isozymes (Martiny-Baron *et al.*, 1993; Le Panse *et al.*, 1994).

As we excluded any modulation of MDR1 gene expression by the bisindolylmaleimide after a 24 h incubation of two different types of MDR cells, i.e. a T-lymphoblastoid CCRF-CEM subline and an adenocarcinoma KB subline (Figure 12), there is no reason to assume an involvement of the PKC isozymes $\alpha$, $\beta$1, $\beta$2, $\gamma$, $\delta$ and $\varepsilon$ in MDR1 gene expression under the conditions applied. Considering the $IC_{50}$ value of 5.8 $\mu$M observed *in vitro* on PKC$\zeta$ preparations (Martiny-Baron *et al.*, 1993), even an involvement of PKC$\zeta$ seems not very likely, because at least some partial MDR1 mRNA down-modulation should be expected at the concentrations applied (7.5 or 10 $\mu$M GF 109203X), if such a link actually exists in the cell types investigated here. It has to be emphasised, however, that our results do not exclude a PKC-mediated impact on MDR1 gene expression becoming relevant as part of a rather complex stress response (Chaudhary and Roninson, 1993) in a transient manner possibly including other factors, by analogy with our observation that only a stimulated c-*fos* promoter activity is suppressed by the PKC inhibitor (Figure 3).

**Figure 12** Effects of GF 109203X on MDR1 gene expression (mean $\pm$ standard deviation, $n=3$) after a 24 h incubation of (**a**) KB 8.5 cells, and (**b**) CCRF VCR 1000 cells at the final concentrations indicated. The cDNA-PCR was performed by increasing the number of PCR cycles. The signal intensities were calculated after normalisation onto the signal intensities obtained by the GAPDH amplimers where 23 PCR cycles were applied throughout, which was proven to be in the exponential range of PCR yield.

The dose–response curves of MDR modulation (Figures 4–8), showing half-maximal effects in the range of 3 $\mu$M or even higher final concentrations of GF 109203X, correspond well to the dose–responses seen in radioligand ([$^3$H]vinblastine sulphate) binding (Figure 6b) or photoaffinity labelling ([$^3$H]azidopine) competition experiments using crude membrane preparations from CCRF ADR 5000 cells. We therefore suggest that the observed MDR modulation by the bisindolylmaleimide PKC inhibitor GF 109203X is essentially caused by direct interaction of the compound with P-gp. Then, the view appears justified that at least the PKC isozymes accordingly inhibited by GF 109203X strongly, i.e. $\alpha$, $\beta$1, $\beta$2, $\gamma$, $\delta$ and $\varepsilon$, do not contribute much to the P-gp-mediated MDR in the cell systems used by us. This also corresponds to the finding published recently (Gupta *et al.*, 1994) that calphostin C devoid of PKC-inhibitory activity because of exclusion of light exposure, modulates MDR in about the same manner as the activated calphostin C. Even staurosporine accordingly binds to P-gp (Sato *et al.*, 1990; Miyamoto *et al.*, 1993). Similar observations were reported for the staurosporine derivative NA-382 (Miyamoto *et al.*, 1993). The recently reported strong MDR-modulating effects (Utz *et al.*, 1994) of another staurosporine derivative (CGP 41251) could also be explained by direct interaction with P-gp.

Our data indicate the need for investigating the direct binding of MDR-modulating compounds to P-gp for evaluation of the virtual mechanisms of action. It might not be surprising that many structurally rather complex compounds usually derived from naturally occurring drugs interact with P-gp.

Nonetheless, we did not investigate the phosphorylation of cellular targets under the influence of GF 109203X as described by Toullec *et al.* (1991). Thus, at present we cannot state whether phosphorylation of P-gp actually occurs in the cell lines investigated here and at which final concentrations the compound GF 109203X might influence in a cellular assay the phosphorylation of P-gp and/or other cellular PKC substrates. Moreover, a contribution to P-gp activity of particular PKC isozymes like PKCζ (as proposed recently by Bates *et al.*, 1993), poorly affected by GF 109203X, cannot be excluded. However, no influence of P-gp phosphorylation on P-gp transport function was observed by Scala *et al.* (1995). Moreover, an altered P-gp in which the serines at positions 661, 667, 671, 675 and 683 were replaced by non-phosphorylatable alanine residues showed neither significant [$^{32}$P]orthophosphate incorporation nor a significantly disturbed transport function (Germann *et al.*, 1996). Altogether, there is no experimental evidence for a major contribution of P-gp phosphorylation to MDR in a variety of cell types and transfectants.

As it was shown previously (Toullec *et al.*, 1991) that the bisindolylmaleimide GF 109203X competes with ATP at PKC, we speculate on an interaction of the drug with the ATP binding site(s) of P-gp as well, which might also be true for the reversal by GF 109203X of the MDR mediated by the newly identified ABC (ATP binding cassette) drug transporter MRP (multidrug resistance associated protein) (Gekeler *et al.*, 1995). From our data it seems obvious, however, that chemosensitisers binding to P-gp with high affinity (such as DNIG) may be particularly valuable for clinical use as MDR modulators. Nonetheless, the bisindolylmaleimide represents a new tool structure possibly binding to a site on P-gp different from the binding site of DNIG, which supposedly acts as an allosteric inhibitor of P-gp according to Ferry *et al.* (1992) and Malkhandi *et al.* (1994).

**Abbreviations**

ATCC, American Type Culture Collection; CAT, chloramphenicol acetyl transferase; cDNA-PCR, complementary DNA polymerase chain reaction; DMSO, dimethylsulphoxide; DNIG, dexniguldipine-HCl; DVER, dexverapamil-HCl; DOX, doxorubicin; GAPDH, glyceraldehyde-3-phosphate dehydrogenase; HEPES, 4-(2-hydroxyethyl)-1-piperazine-ethanesulphonic acid; $IC_{50}$, 50% inhibitory concentration; MDR, multidrug resistance; MDR1, human MDR1 gene; MTT, 3-(4,5-dimethylthiazol-2-yl)-2,5-diphenyl tetrazolium bromide; PBS, phosphate-buffered saline; PDBu, phorbol-12,13-dibutyrate; P-gp, P-glycoprotein; PKC, protein kinase C (EC 2.7.1.37); PMSF, phenylmethylsulphonyl fluoride; STAU, staurosporine; TPA (=PMA), phorbol-12-myristate-13-acetate; VCR, vincristine.

**Acknowledgements**

We gratefully acknowledge and thank C Borchers for help in photoaffinity labelling experiments, and M Giel, S Haas, M Heuser, A Schödl and M Schwarz for excellent technical assistance. We also thank Dr M M Gottesman for providing the cell lines KB 3.1 and KB 8.5 and the MDR1 expression vector pSK1.MDR. Parts of the work were supported by a grant (P-08545-MED) from the Austrian Science Foundation.

### References

AHMAD S AND GLAZER RI. (1993). Expression of the antisense cDNA for protein kinase Cα attenuates resistance in doxorubicin-resistant MCF-7 breast carcinoma cells. *Mol. Pharmacol.*, **43**, 858–862.

AKIYAMA S, FOJO AT, HANOVER JA, PASTAN I AND GOTTESMAN MM. (1985). Isolation and genetic characterization of human KB cell lines resistant to multiple drugs. *Somatic Cell Mol. Genet.*, **11**, 117–126.

BATES SE, LEE JS, DICKSTEIN B, SPOLYAR M AND FOJO AT. (1993). Differential modulation of P-glycoprotein transport by protein kinase inhibition. *Biochemistry*, **32**, 9156–9164.

BECK J, HANDGRETINGER R, DOPFER R, KLINGEBIEL T, NIETHAMMER D AND GEKELER V. (1995*a*). Expression of mdr1, mrp, topoisomerase IIα/β, and cyclin A in primary or relapsed states of acute lymphoblastic leukaemias. *Br. J. Haematol.*, **89**, 356–363.

BECK J, GEKELER V, HANDGRETINGER R AND NIETHAMMER D. (1995*b*). Expression of PKC isozyme and MDR-related genes in primary and relapsed state AML. *Proc. Am. Assoc. Cancer Res.*, **36**, 335.

BOER R, HAAS S AND SCHÖDL A. (1994). Influence of dexniguldipine-HCl on rhodamine-123 accumulation in a multidrug-resistant leukaemia cell line: comparison with other chemosensitizers. *Eur. J. Cancer*, **30A**, 1117–1123.

BORCHERS C, ULRICH W-R, KLEMM K, ISE W, GEKELER V, HAAS S, SCHÖDL A, CONRAD J, PRZYBILSKI M AND BOER R. (1995). B9209–005, an azidoderivative of the chemosensitizer dexniguldipine-HCl photolabels P-glycoprotein. *Mol. Pharmacol.*, **48**, 21–29.

CHAMBERS TC, ZHENG B AND KUO JF. (1992). Regulation by phorbol ester and protein kinase C inhibitors, and by a protein phosphatase inhibitor (okadaic acid), of P-glycoprotein phosphorylation and relationship to drug accumulation in multidrug-resistant human KB cells. *Mol. Pharmacol.*, **41**, 1008–1015.

CHAMBERS TC, POHL J, RAYNOR RL AND KUO JF. (1993). Identification of specific sites in human P-glycoprotein phosphorylated by protein kinase C. *J. Biol. Chem.*, **268**, 4592–4595.

CHAUDHARY PM AND RONINSON IB. (1993). Induction of multidrug resistance in human cells by transient exposure to different chemotherapeutic drugs. *J. Natl Cancer Inst.*, **85**, 632–639.

EPAND RM AND STAFFORD AR. (1993). Protein kinases and multidrug resistance. *Cancer J.*, **6**, 154–158.

FERRY DR, RUSSELL MA AND CULLEN MH. (1992). P-glycoprotein possesses a 1,4-dihydropyridine-selective drug acceptor site which is allosterically coupled to a vinca-alkaloid-selective binding site. *Biochem. Biophys. Res. Commun.*, **188**, 440–445.

GEKELER V, FRESE G, DIDDENS H AND PROBST H. (1988). Expression of a P-glycoprotein gene is inducible in a multidrug-resistant human leukemia cell line. *Biochem. Biophys. Res. Commun.*, **155**, 754–760.

GEKELER V, WEGER S AND PROBST H. (1990). Mdr1/P-glycoprotein gene segments analyzed from various human leukemic cell lines exhibiting different multidrug resistance profiles. *Biochem. Biophys. Res. Commun.*, **169**, 796–802.

GEKELER V, BECK J, NOLLER A, WILISCH A, FRESE G, NEUMANN M, HANDGRETINGER R, EHNINGER G, PROBST H AND NIETHAMMER D. (1994). Drug-induced changes in the expression of MDR-associated genes: investigations on cultured cell lines and chemotherapeutically treated leukemias. *Ann. Hematol.*, **69**, S19–S24.

GEKELER V, BOER R, ISE W, SANDERS KH, SCHÄCHTELE C AND BECK J. (1995). The specific bisindolylmaleimide PKC-inhibitor GF 109203X efficiently modulates MRP-associated multiple drug resistance. *Biochem. Biophys. Res. Commun.*, **206**, 119–126.

GERMANN U, CHAMBERS TC, AMBUDKAR SV, LICHT T, CARDARELLI CO, PASTAN I AND GOTTESMAN MM. (1996). Characterization of phosphorylation-defective mutants of human P-glycoprotein expressed in mammalian cells. *J. Biol. Chem.*, **271**, 1708–1716.

GRUNICKE H, HOFMANN J, UTZ I AND ÜBERALL F. (1994). Role of protein kinases in antitumor drug resistance. *Ann. Hematol.*, **69**, S1–S6.

GUPTA S, PATEL K, SINGH H AND GOLLAPUDI S. (1994). Effect of calphostin C (PKC inhibitor) on daunorubicin resistance in P388/ADR and HL60/AR cells: reversal of drug resistance possibly via P-glycoprotein. *Cancer Lett.*, **76**, 139–145.

HAMADA H, HAGIWARA K-I, NAKAJIMA T AND TSURUO T. (1987). Phosphorylation of the $M_r$ 170 000 to 180 000 glycoprotein specific to multidrug-resistant tumor cells: effects of verapamil, trifluoperazine, and phorbol esters. *Cancer Res.*, **47**, 2860–2865.

HARTENSTEIN JH, ARANDA J, BARTH H, KLEINSCHROTH J, RECK R, RUDOLPH C, TROSTMANN U AND SCHÄCHTELE C. (1993). The design of protein kinase C inhibitors. In *Perspectives in Medicinal Chemistry*, Testa B, Kyburz E, Fuhrer W and Giger R. (eds) pp. 99–118. Verlag Helvetica Chimica Acta: Basle, Switzerland.

HEIKKILÄ J, JALAVA A AND ERIKSSON K. (1993). The selective protein kinase C inhibitor GF 109203X inhibits phorbol ester-induced morphological and functional differentiation of SH-SY5Y human neuroblastoma cells. *Biochem. Biophys. Res. Commun.*, **197,** 1185–1193.

HOFMANN J, GEKELER V, ISE W, NOLLER A, MITTERDORFER J, HOFER S, UTZ I, GOTWALD M, BOER R, GLOSSMANN H AND GRUNICKE H. (1995). Mechanism of action of dexniguldipine-HCl (B8509-035), a new potent modulator of multidrug resistance. *Biochem. Pharmacol.*, **49,** 603–609.

KANE SE, REINHARD DH, FORDIS CM, PASTAN I AND GOTTESMAN MM. (1989). A new vector using the human multidrug resistance gene as a selectable marker enables overexpression of foreign genes in eukaryotic cells. *Gene*, **84,** 439–446.

KIMMIG A, GEKELER V, NEUMANN M, FRESE G, HANDGRETINGER R, KARDOS G, DIDDENS H AND NIETHAMMER D. (1990). Susceptibility of multidrug-resistant human leukemia cell lines to human interleukin 2-activated killer cells. *Cancer Res.*, **50,** 6793–6799.

KOHNO K, SATO S, TAKANO H, MATSUO K AND KUWANO M. (1989). The direct activation of human multidrug resistance gene (mdr1) by anticancer agents. *Biochem. Biophys. Res. Commun.*, **165,** 1415–1421.

KÖNIG H, PONTA H, RAHMSDORF U, BÜSCHER M, SCHÖNTHAL A, RAHMSDORF J AND HERRLICH P. (1989). Autoregulation of fos: the dyad symmetry element as the major target of repression. *EMBO J.*, **8,** 2559–2566.

LE PANSE R, COULOMB B, MITEV V, BOUCHARD B, LEBRETON C AND DUBERTRET L. (1994). Differential modulation of human fibroblast and keratinocyte growth by the protein kinase C inhibitor GF 109203X. *Mol. Pharmacol.*, **46,** 445–451.

MALKHANDI J, FERRY DR, BOER R, GEKELER V, ISE W AND KERR DJ. (1994). Dexniguldipine-HCl is a potent allosteric inhibitor of [$^3$H]vinblastine binding to P-glycoprotein of CCRF ADR-5000 cells. *Eur. J. Pharmacol.*, **288,** 105–113.

MARTINY-BARON G, KAZANIETZ MG, MISCHAK H, BLUMBERG PM, KOCHS G, HUG H, MARMÉ D AND SCHÄCHTELE C. (1993). Selective Inhibition of protein kinase C isozymes by the indolocarbazole Gö 6976. *J. Biol. Chem.*, **268,** 9194–9197.

MIYAMOTO K, INOKO K, WAKUSAWA S, KAJITA S, HASEGAWA T, TAKAGI K AND MASAO K. (1993). Inhibition of multidrug resistance by a new staurosporine derivative, NA-382, *in vitro* and *in vivo*. *Cancer Res.*, **53,** 1555–1559.

MOSMANN TJ. (1983). Rapid colorimetric assay for cellular growth and survival: application to proliferation and cytotoxic assays. *J. Immunol. Methods*, **65,** 55–63.

NEUMANN M, WILISCH A, DIDDENS H, PROBST H AND GEKELER V. (1992). MDR hamster cells exhibiting multiple altered gene expression: effects of DNIG (B859-35), cyclosporin A and buthionine sulfoximine. *Anticancer Res.*, **12,** 2297–2302.

SATO W, YUSA K, NAITO M AND TSURUO T. (1990). Staurosporine, a potent inhibitor of C-kinase, enhances drug accumulation in multidrug-resistant cells. *Biochem. Biophys. Res. Commun.*, **173,** 1252–1257.

SCALA S, DICKSTEIN B, REGIS J, SZALLASI Z, BLUMBERG PM AND BATES SE. (1995). Bryostatin 1 affects P-glycoprotein phosphorylation but not function in multidrug-resistant human breast cancer cells. *Clin. Cancer Res.*, **1,** 1581–1587.

SZALLASI Z, SMITH CB, PETTIT GR AND BLUMBERG PM. (1994). Differential regulation of protein kinase C isozymes by bryostatin 1 and phorbol 12-myristate 13-acetate in NIH 3T3 fibroblasts. *J. Biol. Chem.*, **269,** 2118–2124.

TOULLEC D, PIANETTI P, COSTE H, BELLEVERGUE P, GRAND-PERRET T, AJAKANE M, BAUDET V, BOISSIN P, BOURSIER E, LORIOLLE F, DUHAMEL L, CHARON D AND KIRILOVSKY J. (1991). The bisindolylmaleimide GF 109203X is a potent and selective inhibitor of protein kinase C. *J. Biol. Chem.*, **266,** 15771–15781.

ÜBERALL F, MALY K, EGLE A, DOPPLER W, HOFMANN J AND GRUNICKE HH. (1991). Inhibition of cell proliferation, protein kinase C, and phorbol ester-induced fos expression by the dihydropyridine derivative B859-35. *Cancer Res.*, **51,** 5821–5825.

ÜBERALL F, WERNER-FELMAYER G, SCHUBERT C, GRUNICKE HH, WACHTER H AND FUCHS D. (1994). Neopterin derivatives together with cyclic guanosine monophosphate induces c-fos gene expression. *FEBS Lett.*, **352,** 11–14.

UTZ I, HOFER S, REGENASS U, HILBE W, THALER J, GRUNICKE H AND HOFMANN J. (1994). The protein kinase C inhibitor CGP 41251, a staurosporine derivative with antitumor activity, reverses multidrug resistance. *Int. J. Cancer*, **57,** 104–110.

YU G, AHMAD S, AQUINO A, FAIRCHILD CR, TREPEL JB, OHNO S, SUZUKI K, TSURUO T, COWAN KH AND GLAZER RI. (1991). Transfection with protein kinase C$\alpha$ confers increased multidrug resistance to MCF-7 cells expressing P-glycoprotein. *Cancer Commun.*, **3,** 181–189.

YUSA K AND TSURUO T. (1989). Reversal mechanism of multidrug resistance by verapamil: direct binding of verapamil to P-glycoprotein on specific sites and transport of verapamil outward across the plasma membrane of K562/ADM cells. *Cancer Res.*, **49,** 5002–5006.